# Effects of *ε*‐viniferin, a dehydrodimer of resveratrol, on transepithelial active ion transport and ion permeability in the rat small and large intestinal mucosa

**DOI:** 10.14814/phy2.12790

**Published:** 2016-05-09

**Authors:** Shin‐Ichiro Karaki, Junji Ishikawa, Yuka Tomizawa, Atsukazu Kuwahara

**Affiliations:** ^1^ Laboratory of PhysiologyDepartment of Environmental and Life SciencesUniversity of ShizuokaShizuokaJapan; ^2^ FANCL Research InstituteFANCL CorporationYokohamaJapan

**Keywords:** Epithelial ion transport, intestinal chemosensing, ussing chamber

## Abstract

*ε*‐Viniferin is a dehydrodimer of resveratrol, a polyphenol synthesized in many plants, including grapevine. The present study investigated the effects of *ε*‐viniferin and resveratrol on epithelial secretory and barrier functions in isolated rat small and large intestinal mucosa. Mucosa–submucosa tissue preparations of various segments of the rat large and small intestines were mounted on Ussing chambers, and short‐circuit current (*I*_sc_) and tissue conductance (*G*_t_) were continuously measured. The mucosal addition of *ε*‐viniferin (>10^−5^ mol/L) and resveratrol (>10^−4^ mol/L) to the cecal mucosa, which was the most sensitive region, induced an increase in *I*_sc_ and a rapid phase decrease (P‐1) followed by rapid (P‐2) and broad (P‐3) peak increases in *G*_t_ in concentration‐dependent manners. Mucosal *ε*‐viniferin (10^−4^ mol/L), but not resveratrol (10^−4^ mol/L), increased the permeability of FITC‐conjugated dextran (4 kDa). The mucosal *ε*‐viniferin–evoked changes in *I*_sc_ (Cl^−^ secretion), but not in *G*_t_, were attenuated by a selective cyclooxygenase (COX)‐1 inhibitor and a selective EP_4_ prostaglandin receptor. The mucosal *ε*‐viniferin–evoked increase in *I*_sc_ was partially attenuated, and P‐2, but not P‐1 or P‐3, change in *G*_t_ was abolished by a transient receptor potential cation channel, subfamily A, member 1 (TRPA1) inhibitor. Moreover, the mucosal *ε*‐viniferin concentration‐dependently attenuated the mucosal propionate (1 mmol/L)‐evoked increases in *I*_sc_ and *G*_t_. Immunohistochemical studies revealed COX‐1–immunoreactive epithelial cells in the cecal crypt. The present study showed that mucosal *ε*‐viniferin modulated transepithelial ion transport and permeability, possibly by activating sensory epithelial cells expressing COX‐1 and TRPA1. Moreover, mucosal *ε*‐viniferin decreased mucosal sensitivity to other luminal molecules such as short‐chain fatty acids. In conclusion, these results suggest that *ε*‐viniferin modifies intestinal mucosal transport and barrier functions.

## Introduction

*ε*‐Viniferin is a dehydrodimer of resveratrol (3,5,4′‐trihydroxy‐*trans*‐stilbene), a polyphenol expressed in many plants, including grapevine. Resveratrol has a variety of beneficial effects, including antioxidant (Farghali et al. 2013), anti‐inflammatory (Csiszar 2011), and anticancer effects (Jang et al. 1997; Aggarwal et al. 2004). Additionally, it has been reported that resveratrol oligomers (oligostilbenoids), like *ε*‐viniferin, have a variety of beneficial effects (Xue et al. 2014). However, it is thought that resveratrol oligomers are poorly absorbed in the intestine, unlike resveratrol (Willenberg et al. 2015).

The intestinal epithelia are sensory tissues for luminal molecules (Kaji et al. 2011b; Breer et al. 2012; Furness et al. 2013). Luminal nutrient sensing in the gastrointestinal (GI) tract induces a variety of physiological responses mediated by diverse gut hormones. For example, nutrients stimulate gastrin‐containing enteroendocrine G cells to release gastrin and induce gastric acid secretion. In the large intestine, the most predominant (~100 mmol/L) anions are short‐chain fatty acids (SCFAs), which are enterobacterial fermented products, that induce secretory (Yajima 1988; Karaki and Kuwahara 2011) and contractile responses (Yajima 1985; Mitsui et al. 2005) by activating the luminal side, but not the serosal side. Moreover, we previously reported that a bitter taste (Kaji et al. 2009) and an olfactory compound (Kaji et al. 2011a) evoke secretory responses by activating the luminal side of the GI tract. Therefore, it is possible that some polyphenols, including resveratrol, *ε*‐viniferin, and other oligomers activate the luminal chemosensory system in the GI tract.

It was reported that apical administration of resveratrol stimulates cAMP‐dependent chloride secretion (Cl^−^) in a colonic epithelium‐like monolayer‐cultured cell line, T84 cells, and in the mouse jejunum (Blumenstein et al. 2005). Conversely, it was reported that resveratrol oligomers, including *ε*‐viniferin, inhibited cAMP‐dependent (forskolin‐activated) Cl^−^ secretion (Zhang et al. 2014). Unfortunately, these earlier reports did not investigate the effects of resveratrol or its oligomers on epithelial barrier functions.

Therefore, the aims of the present study were to investigate the mucosal effects of resveratrol and *ε*‐viniferin on epithelial secretory functions and ion permeability in isolated rat small and large intestines.

## Materials and Methods

### Animals and tissue preparation

Male Sprague Dawley (SD) rats (mean ± standard error of the mean [SEM], 335.2 ± 10.3 g, *n *=* *62) were purchased from Japan SLC, Inc. (Hamamatsu, Japan), and were allowed food and water ad libitum before all experiments. The animals were handled and euthanized in accordance with the Guidelines for the Care and Use of Laboratory Animals of the University of Shizuoka, and the study was approved by the University of Shizuoka Animal Usage Ethics Committee. SD rats were anesthetized with isoflurane and decapitated with a guillotine. The terminal ileum, cecum, proximal colon, middle colon, distal colon, and rectum were removed and placed in ice‐cold Krebs–Ringer solution saturated with O_2_ and CO_2_ (see Solutions). Tissues were cut along the mesenteric border, and pinned flat to silicon rubber‐lined Petri dishes with the Krebs–Ringer solution. The smooth muscle layer of the tissue was removed using microscissors under a stereomicroscope, and the mucosa–submucosa preparations were mounted between the halves of Ussing flux chambers (CHM2; World Precision Instruments, Inc., Sarasota, FL) to measure transepithelial ion transport and permeability.

### Ussing chamber experiments

The cross‐sectional area of the mucosa–submucosa preparations in the Ussing flux chamber was 0.64 cm^2^, and both sides were bathed with 10 mL of Krebs–Ringer solution, which was recirculated from a glass circulation reservoir (#5210; World Precision Instruments, Inc.) maintained at 37°C and gassed with 95% O_2_/5% CO_2_. The transepithelial potential difference (*PD*; the serosal electrode served as the reference) was measured using paired Ag–AgCl electrodes (EKV; World Precision Instruments, Inc.) through Krebs–agar bridges, and was clamped at 0 mV by applying a short‐circuit current (*I*_sc_) with another pair of Ag–AgCl electrodes (EKC; World Precision Instruments, Inc.) connected to a voltage‐clamp apparatus (CEZ‐9100; Nihon‐Koden, Tokyo, Japan). The lumen negative electrogenic current represents a positive *I*_sc_ per unit area of tissue (*μ*A/cm^2^). To record tissue conductance (*G*_t_ [mS/cm^2^]), voltage command pulses (10 mV, 3 sec duration) were applied at 1 min intervals, and *G*_t_ was calculated using Ohm's law as the current needed to alter the clamped voltage. The current output was continuously recorded on a data acquisition and analog‐to‐digital conversion system (PowerLab 4/26; ADInstruments, Cattle Hill, Australia). Before the experiments, the tissues were stabilized for 1 h and tissue viability was checked by electrical field stimulation (EFS; 25 V, 5 Hz, 0.5 msec duration for 2 min) using a pair of aluminum foil ribbon electrodes.

### FD4 permeability

The permeability of fluorescein isothiocyanate (FITC)‐conjugated dextran (average molecular weight 4000; FD4) in the mucosa–submucosal tissue preparation was measured under short‐circuit conditions in the Ussing chamber. In this experiment, FD4 (10^−4^ mol/L) was added to the mucosal bathing solution 30 min before the addition of EtOH (10 *μ*L; vehicle control), *ε*‐viniferin (10^−4^ mol/L), and resveratrol (10^−4^ mol/L). Samples (100 *μ*L) were taken from the serosal bathing solution, and fresh Krebs–Ringer (100 *μ*L) was added at −15, 0 15, 30, 45, and 60 min (6 samples). The concentrations of FD4 were determined by measuring the fluorescence intensity of samples and standard solutions using a microplate reader (Varioskan Flash; Thermo Fisher Scientific, Inc., Yokohama, Japan) at emission and excitation wavelengths of 520 nm and 490 nm, respectively. Based on a volume of the serosal bathing solution (*V*_m_) of 10 mL and sample volume (*V*_s_) of 100 *μ*L, the total FD4 mass that crossed the tissue at each time (*M*_*n*_) was:

$$M_{n}=C_{n}\times V_{m}+\left(\sum_{n=1}^{6}C_{n-1}\right)\times V_{s}=C_{n}\times 10\;\text{mL}+\left(\sum_{n=1}^{6}C_{n-1}\right)\times 100\;\mu L$$

where *C*_1_, *C*_2_, … *C*_*n*_ are the sample concentrations at sampling times −15 … 60 min. The FD4 flux (*J* ^m→s^) per unit area (0.64 cm^2^) was calculated as:$J_{n}^{m\to s}=\frac{M_{n+1}-M_{n}}{15\;\text{min}}\times \frac{1}{0.64\;{\text{cm}}^{2}}$where *J*_1_^m→s^, *J*_2_^m→s^, …*J*_*n*‐1_^m→s^ are the FD4 fluxes in each period (−15–0 min, 0–15 min, … 45–60 min). Because the decrease in the mucosal concentration of FD4 during experiments is considered to be negligible, the permeability (*P*_n_^m→s^) of FD4 in each period was calculated by Fick's law as follows:$P_{n}^{m\to s}=\frac{J_{n}^{m\to s}}{C_{m}-C_{s}}≅\frac{J_{n}^{m\to s}}{C_{m}}=\frac{J_{n}^{m\to s}}{10^{-4}\;\text{mol/L}}$where *C*_m_ and *C*_s_ (*C*_m_ ≫ *C*_s_) are the concentrations of FD4 in the mucosal and serosal bathing solutions, respectively. This method was built in reference to a chapter in the textbook, Molecular Biopharmaceutics (Brodin et al. 2009).

### Solutions

The Krebs–Ringer solution contained (in mmol/L) 117 NaCl, 4.7 KCl, 1.2 MgCl_2_, 1.2 NaH_2_PO_4_, 25 NaHCO_3_, and 2.5 CaCl_2_, and was saturated with 95% O_2_/5% CO_2_. The Cl^−^‐free modified Krebs–Ringer solution was prepared by replacing Cl^−^ with an equal concentration of gluconate^−^, and the concentration of Ca‐gluconate_2_ was increased to 8 mmol/L to compensate for the Ca^2+^‐buffering effects of gluconate. The mucosal bathing solutions were supplemented with 5 mmol/L glucose.

### Chemicals

*ε*‐Viniferin, resveratrol, and sodium propionate were purchased from Wako Pure Chemical Industries, Ltd. (Osaka, Japan). Carbachol (CCh), FD4, bumetanide, 5‐nitro‐2‐(3‐phenylpropylamino) benzoic acid (NPPB), atropine, hexamethonium, SC‐560, and NS‐398 were purchased from Sigma (St. Louis, MO). TTX and HC030031 were purchased from Tocris (Ellisville, MO). Piroxicam was purchased from Biomol Research Laboratories (Plymouth Meeting, PA). 4,4′‐Diisothiocyanatostilbene‐2,2′‐disulfonic acid (DIDS) was purchased from ANA SPEC (Fremont, CA). AH‐6809 was purchased from Cayman Chemical (Ann Arbor, MI). ONO‐8713, ONO‐AE3‐208, and ONO‐AE3‐240 were kind gifts from ONO Pharmaceutical Co., Ltd. (Osaka, Japan).

*ε*‐Viniferin and resveratrol were dissolved in ethanol (EtOH) just before use. TTX was dissolved in citrate buffer (pH 4.8) and stored at −20°C until use. CCh, FD4, propionate, atropine, and HEX were dissolved in distilled water, and stored at 4°C until use. All of the other chemicals were dissolved in DMSO and stored at −20°C until use.

### Immunohistochemistry

The isolated cecal tissues were immediately frozen with Tissue‐Tek optimal cutting temperature compound (Sakura Finetek Japan Co., Ltd., Tokyo, Japan) in a −75°C acetone bath (Histo‐Tek Pino; Sakura Finetek Japan Co., Ltd.). The tissues were cut into 4‐*μ*m‐thick sections on a cryostat (CM1100; Leica Microsystems, Weltzlar, Germany). The sections were placed on glass slides, fixed in 100% methanol at −20°C for 10 min, and air‐dried under a cold blower for 30 min. The sections were incubated with 10% normal donkey serum and 1% Triton X‐100 in phosphate‐buffered saline (PBS) at room temperature for 1 h to block nonspecific antibody binding. The sections were incubated with goat anticyclooxygenase (COX)‐1 antibody (×500 dilution; sc‐1754; Santa Cruz Biotechnology, Inc., Santa Cruz, CA) in PBS containing 0.3% Triton X‐100 at 4°C overnight. After washing in PBS (3 × 10 min), the sections were incubated with donkey anti‐goat IgG conjugated with Alexa594 (×500 dilution; A11058; Life Technologies, Carlsbad, CA) at room temperature for 1 h. The sections were washed in PBS (3 × 10 min), and coverslips were mounted on the glass slides with mounting medium (DakoCytomation, Glostrup, Denmark). Immunoreactivity for COX‐1 was visualized using a fluorescence microscope (Axio Observer Z1; Carl Zeiss, Oberkochen, Germany), and the images were captured using a cooled charge‐coupled device digital camera and digital imaging software (AxioCam and AxioVision; Carl Zeiss).

### Data analysis and statistics

All physiologic data in the present study are expressed as the mean ± SEM. Multiple comparisons were made using Dunnett's test and Tukey's test for each group relative to the control group and among all combinations of groups, respectively. Paired *t* tests were used to determine the statistical significance between two groups because some tissue preparations from the same animals were used in both experimental groups. In all analyses, *P*s < 0.05 were considered statistically significant.

## Results

### Effects of mucosal and serosal *ε*‐viniferin and resveratrol on *I*_sc_ and *G*_t_ in the mucosa–submucosa preparations of rat cecum

**Figure 1:**
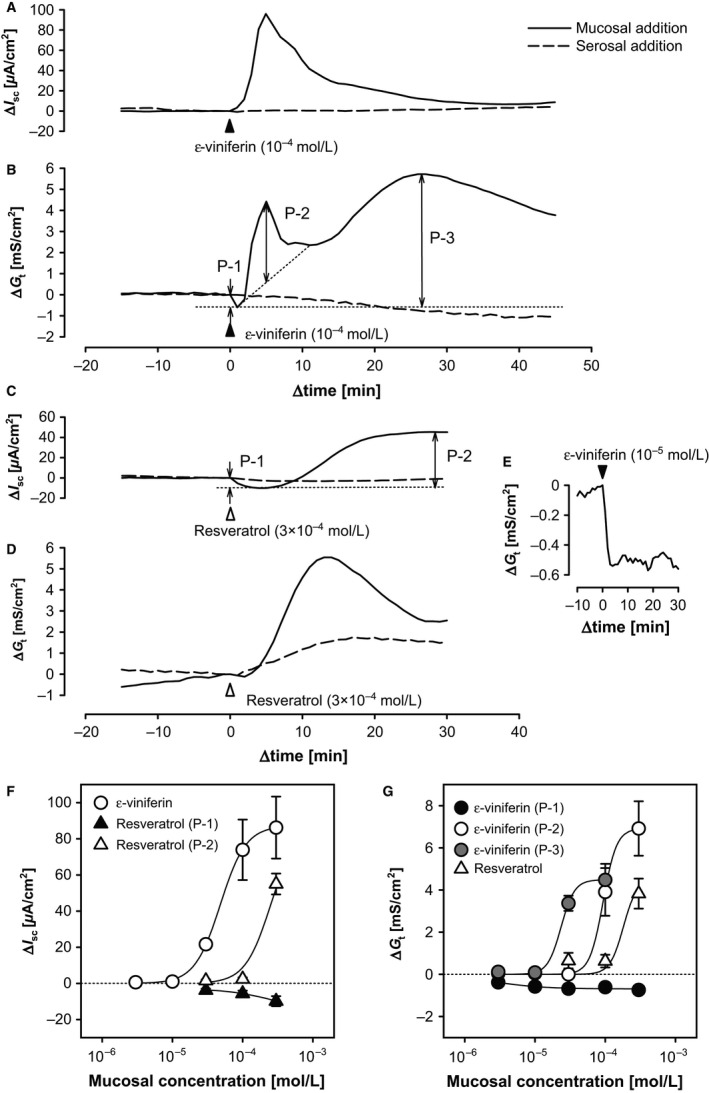
Concentration‐dependent effects of mucosal and serosal *ε*‐viniferin, and mucosal resveratrol on *I*_sc_ and *G*_t_ in the rat cecum. Mucosa–submucosal tissue preparations of rat cecum were mounted on Ussing chambers, and *I*_sc_ and *G*_t_ were continuously recorded. Various concentrations of *ε*‐viniferin (3 × 10^−6^ to 3 × 10^−4^ mol/L) and resveratrol (3 × 10^−5^ to 3 × 10^−4^ mol/L) were added to the mucosal bath at 0 min (Δ). *ε*‐Viniferin (10^−4^ mol/L) was also added to the serosal bath at 0 min. Representative traces from −15 min to 45 min showing the effects of 10^−4^ mol/L mucosal and serosal *ε*‐viniferin (A and B) and 3 × 10^−4^ mol/L mucosal resveratrol (C and D) on Δ*I*_sc_ (A and C) and Δ*G*_t_ (B and D) relative to the values at 0 min. A representative trace showing the effects of a lower concentration (10^−5^ mol/L) of mucosal *ε*‐viniferin on Δ*G*_t_ is shown in (E). The mean peak changes in *I*_sc_ (F) and *G*_t_ (G) were plotted on semi‐log graphs. Data are expressed as the mean ± SEM (*n *=* *4–6).

In the rat cecal mucosa–submucosal preparations, mucosal, but not serosal, addition of *ε*‐viniferin (bath concentration, 10^−4^ mol/L) evoked a monophasic increase in *I*_sc_ (Fig. 1A; ∆*I*_sc_: 73.93 ± 16.75 *μ*A/cm^2^, ∆time: 5.68 ± 0.52 min) and triphasic changes in *G*_t_ (Fig. 1B; P‐1: ∆*G*_t_ −0.61 ± 10.07 mS/cm^2^, ∆time 1.8 ± 0.2 min; P‐2: ∆*G*_t_ 3.91 ± 1.13 mS/cm^2^, ∆time: 5.6 ± 0.2 min; P‐3: ∆*G*_t_ 4.48 ± 0.76 mS/cm^2^, ∆time 20.6 ± 2.3 min; *n *=* *5). In contrast, mucosal addition of 10^−4^ mol/L resveratrol evoked very small changes in *I*_sc_ and *G*_t_ (Fig. 1E and C), while mucosal, but not serosal, addition of 3 × 10^−4^ mol/L resveratrol evoked biphasic changes in *I*_sc_ (Fig. 1C; P‐1: ∆*I*_sc_ −9.84 ± 2.76 *μ*A/cm^2^, ∆time 3.55 ± 0.42 min; P‐2: ∆*I*_sc_ 54.98 ± 5.76 *μ*A/cm^2^, ∆time 24.62 ± 4.77 min; *n *=* *4) and *G*_t_, likely via *ε*‐viniferin–evoked P‐1 (∆*G*_t_ −0.25 ± 0.09 mS/cm^2^, ∆time 2.3 ± 0.5 min) and P‐3 (∆*G*_t_ 3.83 ± 0.71 mS/cm^2^, ∆time 16.8 ± 1.7 min) (*n *=* *4; Fig. 1D). At a lower concentration (10^−5^ mol/L), *ε*‐viniferin evoked a small, but long‐lasting decrease in *G*_t_, as shown in Figure 1E. Mucosal or serosal addition of the vehicle, EtOH (10 *μ*L), did not affect *I*_sc_ or *G*_t_.

Various concentrations of *ε*‐viniferin (3 × 10^−6^ to 3 × 10^−4^ mol/L) and resveratrol (10^−5^ to 3 × 10^−4^ mol/L) were added to the mucosal bathing solution, and the mean changes in *I*_sc_ (Fig. 1E) and *G*_t_ (Fig. 1F) were plotted and fitted to the Hill equation. The half maximal (50%) effective concentration (*EC*_50_) of the mucosal *ε*‐viniferin–evoked ∆*I*_sc_ was 4.81 × 10^−5^ mol/L, the maximum effect (*E*_max_) was 87.19 *μ*A/cm^2^, and the Hill coefficient value (*n*_H_) was 2.37 (coefficient of determination [*R* ^2^] = 1.000). At 10^−5^ mol/L or lower concentrations, mucosal *ε*‐viniferin only evoked a decrease in *G*_t_, which was long‐lasting. The *EC*_50_ of the mucosal *ε*‐viniferin–evoked P‐1 ∆*G*_t_ was 2.53 × 10^−6^ mol/L, *E*_max_ was −0.69 mS/cm^2^, and *n*_H_ was 1.22 (*R* ^2^ = 0.887). Although it is unclear whether the P‐2 ∆*G*_t_ evoked by 3 × 10^−4^ mol/L *ε*‐viniferin was at the maximum effective concentration, the *EC*_50_ of ∆*G*_t_ was calculated to be 9.44 × 10^−5^ mol/L, when we assumed that the *E*_max_ was the mean value for ∆*G*_t_ at 3 × 10^−4^ mol/L *ε*‐viniferin (6.92 mS/cm^2^) and that the *n*_H_ was same as the that for P‐3 described below (*R* ^2^ = 1.000). Therefore, the *EC*_50_ for mucosal *ε*‐viniferin–evoked P‐3 ∆*G*_t_ was calculated to be 2.35 × 10^−5^ mol/L, the *E*_max_ of mucosal *ε*‐viniferin–evoked P‐3 ∆*G*_t_ was 4.49 mS/cm^2^, and nH was 4.53 (*R* ^2^ = 0.999).

The resveratrol‐evoked P‐1 and P‐2 ∆*I*_sc_ did not seem to reach the maximum concentrations, but the *EC*_50_ of P‐2 ∆*I*_sc_ was 2.52 × 10^−4^ mol/L (*R* ^2^ = 0.975) when we assumed that *E*_max_ and *n*_H_ were the same as those for *ε*‐viniferin–evoked ∆*I*_sc_. Likewise, the resveratrol‐evoked increase in *G*_t_ did seem to reach the maximum concentration, but the *EC*_50_ was 1.82 × 10^−4^ mol/L (*R* ^2^ = 0.912) when we assumed the *E*_max_ and *n*_H_ were the same as those for *ε*‐viniferin–evoked P‐3 ∆*G*_t_.

Although *ε*‐viniferin may evoke more potent responses at concentrations of >3 × 10^−4^ mol/L, *ε*‐viniferin at these concentrations evoked unlimited continuous increases in *G*_t_. This indicates that *ε*‐viniferin at concentrations >3 × 10^−4^ mol/L may have cytotoxic effects because the epithelial barrier function was thought to have failed. Thus, *ε*‐viniferin was used at 10^−4^ mol/L in the following experiments.

### Segmental differences in the effects of mucosal *ε*‐viniferin in the rat small and large intestines

**Figure 2:**
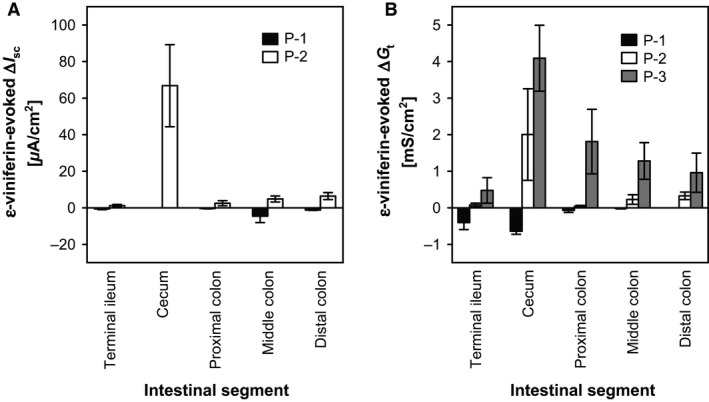
Segmental differences in the *ε*‐viniferin–evoked changes in *I*_sc_ and *G*_t_. Mucosa–submucosal tissue preparations of the rat terminal ileum, cecum, proximal colon, middle colon, and distal colon were mounted on Ussing chambers. *ε*‐Viniferin (10^−4^ mol/L) was added to the mucosal bathing solution, and the peak changes in *I*_sc_ (A) and *G*_t_ (B) were measured. (A) *ε*‐Viniferin evoked a decrease in the first phase (P‐1) and then an increase in the second phase (P‐2) in *I*_sc_. (B) *ε*‐Viniferin evoked a decrease (P‐1), rapid increase (P‐2), and a slow increase (P‐3) in *I*_sc_. Data are expressed as the mean ± SEM (*n *=* *4).

The effects of mucosal *ε*‐viniferin (10^−4^ mol/L) on *I*_sc_ and *G*_t_ were measured and compared among the rat terminal ileum, cecum, proximal colon, middle colon, and distal colon. Mucosal addition of *ε*‐viniferin (10^−4^ mol/L) had weak effects on *I*_sc_ in the intestinal tissues, except for the cecum (Fig. 2A). Regarding *G*_t_, the *ε*‐viniferin (10^−4^ mol/L)–evoked increase was most potent in the cecum and the magnitude of the increase gradually decreased from the proximal to distal segments of the large intestine (Fig. 2B). Thus, the other experiments in the present study were performed using the rat cecum.

### Effects of mucosal *ε*‐viniferin and resveratrol on FD4 permeability in the rat cecum

To investigate the effects of mucosal *ε*‐viniferin and resveratrol on the barrier function against a nonionic macromolecule, the effects of mucosal *ε*‐viniferin and resveratrol on the transepithelial permeability of FD4 from the mucosal to serosal side (FD4 *P* ^m→s^) were measured in terms of *G*_t_. Basal *I*_sc_ was 18.07 ± 1.62 *μ*A/cm^2^, basal *G*_t_ was 11.15 ± 0.41 mS/cm^2^, and basal FD4 *P* ^m→s^ was 1.26 ± 0.20 × 10^−7^ cm/s (*n *=* *20).

Mucosal addition of *ε*‐viniferin evoked an increase in *I*_sc_, and the mean *I*_sc_ reached its maximum at 7 min (Δ*I*_sc_: 83.56 ± 17.12 *μ*A/cm^2^, *n *=* *8). The *G*_t_ first decreased at 3 min (P‐1 Δ*G*_t_: −0.45 ± 0.05 mS/cm^2^, *n *=* *8), the P‐2 peak occurred at 8 min (P‐2 Δ*G*_t_: 4.00 ± 0.82 mS/cm^2^, *n *=* *8), and the P‐3 peak occurred at 26 min (P‐3 Δ*G*_t_: 3.70 ± 0.84 mS/cm^2^, *n *=* *8). FD4 *P* ^m→s^ increased, and reached a maximum (3.98 ± 0.95 × 10^−7^ cm/s, *n *=* *8) at 30–45 min after mucosal addition of *ε*‐viniferin. This value was significantly greater (*P *<* *0.05 by Dunnett's test) than the FD4 *P* ^m→s^ after the addition of the vehicle control (EtOH; 0.138 ± 0.025 × 10^−6^ cm/s, *n *=* *7) at 30–45 min.

After the mucosal addition of resveratrol (10^−4^ mol/L), *I*_sc_ decreased initially (P‐1 Δ*I*_sc_: −4.24 ± 2.06 *μ*A/cm^2^, *n *=* *6, at 5 min), and then slowly increased (P‐2 Δ*I*_sc_: 7.40 ± 4.31 *μ*A/cm^2^, *n *=* *6, at 37 min). *G*_t_ slowly increased (Δ*G*_t_: 1.06 ± 0.22 mS/cm^2^, *n *=* *6, at 17 min). However, FD4 permeability did not significantly change.

### Identification of ion species involved in the *ε*‐viniferin–evoked increase in *I*_sc_ and *G*_t_ in the rat cecum

To identify the ion species involved in the *ε*‐viniferin–induced increase in *I*_sc_, the bathing solutions in the serosal, mucosal, and both chambers were replaced with Cl^−^‐free/gluconate solution. Before mounting the tissues, the fluid resistances of the normal solution and Cl^−^‐free solution in each chamber were measured (normal: 69.5 ± 1.0 Ω; Cl^−^‐free: 114.7 ± 0.9 Ω; *n *=* *3). All preparations were bathed with normal solution at first. After the bathing solutions of the serosal, mucosal, or both sides were replaced with Cl^−^‐free solution, and the compensations of fluid resistance were reset after changing the solutions. Basal *I*_sc_ significantly increased in the serosal Cl^−^‐free solution, but decreased in the mucosal Cl^−^‐free solution (Table 1). In contrast, the basal *G*_t_s were not significantly different between the Cl^−^‐free and sham control tests for each side (Table 1). The negative basal *I*_sc_ in the mucosal Cl^−^‐free solution and the positive basal *I*_sc_ in the serosal Cl^−^‐free solution are considered to be due to the liquid junction potentials between agar bridges and bathing solution.

**Table 1:** Electrophysiological parameters of the rat cecal mucosa–submucosa preparations mounted on Ussing chambers, bathed with normal Krebs–Ringer solution with Cl^−^‐free solutions on the serosal, mucosal, and both sides of the chamber

Krebs‐Ringer solution	Basal *I*_sc_	Basal *G*_t_	*n* (tissue)
Normal (all tissues)	15.15 ± 0.53	12.66 ± 0.16	239
Normal (sham)	11.56 ± 1.02^a^	9.96 ± 0.74	4
Mucosal Cl^−^ free	−39.82 ± 4.90^b^	15.50 ± 3.56	4
Serosal Cl^−^ free	79.72 ± 6.85^c^	12.26 ± 1.73	4
Both sides Cl^−^ free	20.44 ± 0.68^a^	17.56 ± 2.99	4

Basal *I*_sc_ and *G*_t_ values.

*Sham* indicates that normal solutions of both sides of the Ussing chambers were replaced with fresh normal solution. Different superscript letters indicate statistically significant differences (*P *<* *0.001, Tukey's test) among the normal solution and Cl^−^‐free solutions on the serosal, mucosal, and both sides.

The *ε*‐viniferin (10^−4^ mol/L)–evoked increases in *I*_sc_ were almost completely abolished for Cl^−^‐free solution on the serosal and both sides, but not the mucosal side (*P *<* *0.01 by Dunnett's test, *n *=* *4; Fig. 4A). These results indicate that the mucosal *ε*‐viniferin–evoked increase in *I*_sc_ is due to electrogenic Cl^−^ secretion. Moreover, with serosal Cl^−^‐free solution, mucosal *ε*‐viniferin evoked a decrease in *I*_sc_ (−5.55 ± 1.66 *μ*A/cm^2^; Fig. 4A). Serosal and both side Cl^−^‐free solutions led to unlimited increases in *G*_t_, which made it difficult or impossible to measure the mucosal *ε*‐viniferin–evoked changes in *G*_t_.

To confirm that mucosal *ε*‐viniferin evoked Cl^−^ secretion, a Na^+^‐K^+^‐2Cl^−^ (NKCC) transporter inhibitor, bumetanide (10^−4^ mol/L), was added to the serosal bathing solution. Alternatively, a Ca^2+^‐dependent channel blocker, DIDS (10^−4^ mol/L), or a cAMP‐dependent Cl^−^ channel (especially cystic fibrosis transmembrane regulator [CFTR]) blocker, NPPB (10^−5^ to 10^−4^ mol/L), were added to the mucosal bathing solution before the mucosal addition of *ε*‐viniferin. Bumetanide (10^−4^ mol/L) and NPPB (≥5 × 10^−5^ mol/L) significantly attenuated the *ε*‐viniferin–evoked increase in *I*_sc_, but not DIDS (Fig. 5A). Although bumetanide attenuated the *ε*‐viniferin–evoked increase in *I*_sc_, it did not attenuate the *G*_t_ responses (Fig. 5B). In contrast, NPPB (5 × 10^−5^ mol/L) enhanced the *ε*‐viniferin–evoked P‐1 *G*_t_ reduction and nearly abolished the P‐3 *G*_t_ change (Fig. 5B). The *ε*‐viniferin–evoked changes in *G*_t_ in the presence of 10^−4^ mol/L NPPB were not measured because *G*_t_ gradually increased after the addition of 10^−4^ mol/L NPPB.

### Effects of mucosal resveratrol on *I*_sc_, *G*_t_, and *ε*‐viniferin–evoked responses in the rat cecum

Although mucosal addition of 10^–4^ mol/L resveratrol evoked only a weak decrease in P‐1 Δ*I*_sc_ (−5.66 ± 1.61 *μ*A/cm^2^) and an increase in Δ*G*_t_ (0.63 ± 0.30 mS/cm^2^) (Fig. 6; *n *=* *4), resveratrol at 3 × 10^−4^ mol/L elicited *I*_sc_ and *G*_t_ responses, which were similar to those induced by 10^−4^ mol/L *ε*‐viniferin (Fig. 1E and F). We hypothesized that the lower concentration of resveratrol might have an antagonistic function to *ε*‐viniferin. To investigate this hypothesis, *ε*‐viniferin (10^−4^ mol/L) was added to the mucosal bathing solution 30 min after mucosal addition of resveratrol (at 0 to 3 × 10^−4^ mol/L), and the changes in *I*_sc_ and *G*_t_ were measured. In this experiment, resveratrol significantly attenuated the *ε*‐viniferin–evoked changes in *I*_sc_ and *G*_t_ in concentration‐dependent manners (Fig. 6). Mucosal resveratrol at 10^−4^ mol/L significantly attenuated the mucosal *ε*‐viniferin (10^−4^ mol/L)–evoked increase in *I*_sc_ to 26.07 ± 4.12% of the control value (*P *<* *0.05, *n *=* *4) and P‐3 ∆*G*_t_ to 29.31 ± 8.20% of the control value (*P *<* *0.01, *n *=* *5). When the mucosal bathing solution contained 3 × 10^−4^ mol/L resveratrol, the administration of *ε*‐viniferin (10^−4^ mol/L) decreased *I*_sc_ (P‐1 Δ*I*_sc_: −8.60 ± 0.71 *μ*A/cm^2^, *P *<* *0.001, *n *=* *4) and abolished the P‐2 response. However, there were no statistically significant differences because the P‐2 response in the control conditions was weak and varied considerably between tests. Moreover, in the presence of 3 × 10^−4^ mol/L resveratrol, *ε*‐viniferin (10^−4^ mol/L) caused a continuous increase in *G*_t_, as shown in Figure 6C, so P‐3 ∆*G*_t_ could not be determined.

### Effects of a transient receptor potential cation channel, subfamily A, member 1 (TRPA1) inhibitor on mucosal *ε*‐viniferin–evoked responses in the rat cecum

In our previous study, we reported that mucosal administration of a pungent principle, allyl isothiocyanate (AITC), evoked anion secretion by activating TRPA1 channels (Kaji et al. 2012). Therefore, we investigated whether the *ε*‐viniferin–evoked changes in *I*_sc_ and *G*_t_ were mediated via TRPA1 channels, like those evoked by AITC. In this setting, a TRPA1 inhibitor, HC030031 (10^−4^ mol/L), significantly reduced the *ε*‐viniferin–evoked increase in *I*_sc_ from 60.95 ± 10.70 *μ*A/cm^2^ to 38.04 ± 8.02 *μ*A/cm^2^ (*P *<* *0.05, paired *t* test, *n *=* *4; Fig. 7B), and the peak time after the addition of *ε*‐viniferin was significantly delayed from 6.71 ± 0.93 min to 14.68 ± 2.05 min (*P *<* *0.05, paired *t* test, *n *=* *4; Fig. 7A). For *G*_t_, the *ε*‐viniferin–evoked P‐1 and P‐3 responses were not affected, but the P‐2 response was almost completely abolished by HC030031, as shown in Figure 7A and B (*P *<* *0.01, *n *=* *4).

### Lack of effects of neural blockade and cholinergic antagonists, and the involvement of COX activity in the mucosal *ε*‐viniferin–evoked *I*_sc_ and *G*_t_ responses in the rat cecum

The neural reflex pathways in the submucosal plexus in the gut wall play important roles in luminal stimuli‐evoked secretory responses in the intestinal mucosa (Cooke 1998; Karaki and Kuwahara 2004). Thus, we investigated whether neural and cholinergic pathways are involved in the *ε*‐viniferin–induced *I*_sc_ and *G*_t_ responses by adding TTX (a neural blocker; 10^−6^ mol/L), HEX (a nicotinic ACh receptor antagonist; 10^−4^ mol/L), or atropine (a muscarinic ACh receptor antagonist) to the serosal bathing solution 30 min before the addition of *ε*‐viniferin (10^−4^ mol/L). However, TTX, HEX, and atropine did not affect the *ε*‐viniferin–evoked changes in *I*_sc_ and *G*_t_ (*n *=* *4, Fig. 8A, B).

In our previous studies, some mucosal stimulants including a bitter tastant (6‐*n*‐propyl‐2‐thiouracil) (Kaji et al. 2009), an odorant (thymol) (Kaji et al. 2011a), and a pungent principle (AITC) (Kaji et al. 2012) increased *I*_sc_, and these effects were mediated by COX because piroxicam attenuated these effects. Therefore, we hypothesized that the *ε*‐viniferin–evoked *I*_sc_ and *G*_t_ responses were dependent on COX activity. To test this hypothesis, a nonselective COX inhibitor (piroxicam; 10^−5^ mol/L), a selective COX‐1 inhibitor (SC‐560; 10^−5^ mol/L), a selective COX‐2 inhibitor (NS‐398; 10^−5^ mol/L), or a combination of SC‐560 and NS‐398 were added to the serosal bathing solution 30 min before the mucosal addition of *ε*‐viniferin (10^−4^ mol/L). Piroxicam, SC‐560, and the combination of SC‐560 and NS‐398, but not NS‐398 alone, significantly attenuated the *ε*‐viniferin–evoked increase in *I*_sc_ from the control value of 59.40 ± 10.53 *μ*A/cm^2^ (*n *=* *6) to 13.33 ± 2.79 *μ*A/cm^2^ with piroxicam (*n *=* *4; *P *<* *0.01, Dunnett's test), to 23.10 ± 6.36 *μ*A/cm^2^ with SC‐560 (*n *=* *6; *P *<* *005, Dunnett's test), and 22.92 ± 7.70 *μ*A/cm^2^ with the combination of SC‐560 and NS‐398 (Fig. 8B). These results indicate that the *ε*‐viniferin–evoked increase in *I*_sc_ is mediated by COX‐1 production of prostaglandin (P)G. In contrast, piroxicam, SC‐560, and NS‐398 did not significantly affect the *ε*‐viniferin–evoked changes in *G*_t_ (Fig. 8B).

### Effects of selective prostaglandin E_2_ receptor antagonists on mucosal *ε*‐viniferin–evoked responses in the rat cecum

To determine which PG receptor subtype mediates the *ε*‐viniferin–evoked increase in *I*_sc_, a selective EP_1_ receptor antagonist (ONO‐8713; 10^−5^ mol/L), an EP_1_ and EP_2_ receptor antagonist (AH‐6809; 10^−5^ mol/L), a selective EP_3_ receptor antagonist (EP_3_ > EP_4_; ONO‐AE3‐240; 10^−6^ to 10^−5^ mol/L), or a selective EP_4_ receptor antagonist (EP_4_ > EP_3_; ONO‐AE3‐208; 10^−7^ to 10^−5^ mol/L) were added to the serosal bathing solution 30 min before the mucosal addition of *ε*‐viniferin (10^−4^ mol/L). In this experiment, neither ONO‐8713 nor AH‐6809 affected the *ε*‐viniferin–evoked increase in *I*_sc_, but ONO‐AE3‐208 at concentrations ≥10^−6^ mol/L significantly attenuated the *ε*‐viniferin–evoked increase in *I*_sc_ (from 84.47 ± 18.14 *μ*A/cm^2^ to 17.81 ± 10.21 *μ*A/cm^2^ at 10^−6^ mol/L; *P *<* *0.05, *n *=* *4; Fig. 9A). These results indicate that the *ε*‐viniferin–evoked increase in *I*_sc_ is mediated by EP_4_ receptors.

### Inhibitory effects of mucosal *ε*‐viniferin on the mucosal propionate‐evoked *I*_sc_ response

Short‐chain fatty acids, including acetate (two carbons), propionate (three carbons), and butyrate (four carbons), are the predominant anions in the large intestine and exist at concentrations of ≥100 mmol/L. They are produced by bacterial fermentation of indigestible dietary fibers and oligosaccharides. Mucosal SCFAs (with a potency order of propionate ≥ butyrate ≫ acetate) are known to stimulate the large intestinal mucosa to secrete transepithelial anions (Yajima 1988; Karaki and Kuwahara 2011). In our previous studies, luminal thymol and AITC attenuated propionate‐evoked anion secretion in the rat colon (Kaji et al. 2011a, 2012). Therefore, we hypothesized that *ε*‐viniferin would attenuate the mucosal propionate‐evoked secretory responses. To confirm this hypothesis, mucosal propionate (10^−3^ mol/L)‐evoked *I*_sc_ responses were measured 30 min after the addition of *ε*‐viniferin at a variety of concentrations, as described earlier.

In the absence of *ε*‐viniferin, propionate (10^−3^ mol/L) transiently increased *I*_sc_ (Δ*I*_sc_: 222.33 ± 17.45 *μ*A/cm^2^; Fig. 11A) and *G*_t_ (Δ*G*_t_: 6.39 ± 0.67 mS/cm^2^; Fig. 11B) (*n *=* *6). However, the mucosal propionate (10^−3^ mol/L)‐evoked increases in *I*_sc_ and *G*_t_ were attenuated by mucosal *ε*‐viniferin in a concentration‐dependent manner at *ε*‐viniferin concentrations ranging from 3 × 10^−6^ to 3 × 10^−4^ mol/L (Fig. 11). Pretreatment with 3 × 10^−4^ mol/L *ε*‐viniferin nearly abolished the propionate response. The concentration–response curves of *ε*‐viniferin for inhibiting the propionate‐evoked *I*_sc_ and *G*_t_ responses were drawn by fitting the data to the inhibitory Hill equation, as described in Figure 11. In this analysis, the half‐maximal inhibitory concentration (*IC*_50_) of *ε*‐viniferin for Δ*I*_sc_ was 4.12 × 10^−5^ mol/L and the *n*_H_ was 2.11 (*R* ^2^ = 0.998). For *G*_t_, the *IC*_50_ was 2.68 × 10^−5^ mol/L and *n*_H_ was 1.68 (*R* ^2^ = 0.985), where 119.4%, which was the mean value of the propionate‐evoked *G*_t_ in 3 × 10^−6^ mol/L *ε*‐viniferin, was used as the value for the maximal effect.

### Immunohistochemistry for COX‐1 in the rat cecum

It was hypothesized that luminal *ε*‐viniferin might stimulate some epithelial sensory cells expressing COX‐1, because luminal *ε*‐viniferin evoked *I*_sc_ and *G*_t_ responses, but serosal *ε*‐viniferin did not. An earlier study revealed that COX‐1–expressing epithelial cells are scattered throughout the crypts in the rat large intestine (Shao et al. 1999). However, there are no previous reports describing COX‐1 expression in the rat cecum. Thus, we performed immunohistochemical analysis of COX‐1 expression in the rat cecum. Figure 10 shows the images of COX‐1 immunoreactivity in the rat cecal crypt. COX‐1 immunoreactivity was detected on scattered cells in the crypt epithelial cells (Fig. 10).

## Discussion

The results of the present study show that *ε*‐viniferin, a dehydrodimer of resveratrol, stimulated the rat intestinal mucosa, especially the cecal mucosa, from its luminal side, whereas a 10 times higher concentration of resveratrol was needed to evoke the same effect. Mucosal *ε*‐viniferin had three major effects: (1) induce transepithelial electrogenic Cl^−^ secretion by activating EP_4_ receptors via PGs produced by COX‐1; (2) elicit a rapid decrease and a sustained increase in transepithelial ion permeability; and (3) increase transepithelial permeability of a nonionic macromolecule, FD4. In addition, mucosal *ε*‐viniferin inhibited mucosal propionate‐evoked Cl^−^ secretion. These results indicate that *ε*‐viniferin stimulates the intestinal epithelium from the luminal side and effects mucosal barrier functions.

### Mucosal *ε*‐viniferin– and resveratrol‐evoked changes in *I*_sc_ and *G*_t_

Mucosal *ε*‐viniferin at concentrations ≥10^−5^ mol/L elicited a concentration‐dependent monophasic positive change in *I*_sc_ and triphasic changes in *G*_t_, which comprised an abrupt decrease (P‐1) followed by fast (P‐2) and sustained (P‐3) increases in the rat cecal mucosa, whereas serosal administration of 10^−4^ mol/L *ε*‐viniferin did not (Fig. 1A and B). On the other hand, 10^−4^ mol/L resveratrol evoked little, but 3 × 10^−4^ mol/L resveratrol evoked biphasic changes in *I*_sc_, which included a weak negative ∆*I*_sc_ (P‐1) and a sustained positive ∆*I*_sc_ (P‐2), and a single sustained increase in *G*_t_. In contrast, serosal administration of 3 × 10^−4^ mol/L resveratrol did not affect *I*_sc_ and a small decrease (P‐1) and increase (P‐2) in *G*_t_ (Fig. 1C and D) were observed. These results suggest that *ε*‐viniferin stimulates apical targets on the epithelium with a potency 10 times greater than that of resveratrol. Moreover, the first negative ∆*I*_sc_ evoked by *ε*‐viniferin was apparently masked by the positive phase ∆*I*_sc_. In fact, the *ε*‐viniferin–evoked negative ∆*I*_sc_ was observed when the positive ∆*I*_sc_ was inhibited, as in the serosal Cl^−^‐free condition described below.

At lower concentrations (≤10^−5^ mol/L), *ε*‐viniferin evoked the long‐lasting decreases in P‐1 *G*_t_, as shown in Figure 1E. This suggested that, at higher concentration (>3 × 10^−5^ mol/L), the *ε*‐viniferin–evoked changes in *G*_t_ consisted of an abrupt and long‐lasting decrease in ion permeability and subsequent increases in ion permeability. At lower concentration, *ε*‐viniferin may enhance epithelial barrier function, and at higher concentrations *ε*‐viniferin may further enhance ionic and nonionic permeability (as mentioned below) to induce secretory and inflammatory functions as part of a host defense mechanism.

### Segmental differences in the effects of mucosal *ε*‐viniferin

The most potent effects of *ε*‐viniferin in terms of the *I*_sc_ and *G*_t_ responses occurred in the cecal mucosa (Fig. 2). The physiologic meaning of these findings might be related to the primary function of the cecum as a fermentation tank. The gut microbiota in adult humans, especially in the colon, consists of more than 100 trillion microbes of at least 400 species (Bourlioux et al. 2003). The cecum in rodents, including rats, has not atrophied like that in humans, and is the major reservoir of microbiota in the gut. Thus, a variety of compounds produced by fermentation, including organic acids, alcohols, aldehydes, and phenols (Garner et al. 2007), are thought to accumulate in the cecal lumen owing to the activity of cecal microbiota. We hypothesize that the cecal epithelial membrane monitors the luminal fermentation conditions by sensing the luminal content. This is because maintaining adequate luminal conditions for microbiota within the major fermentation site is likely to be extremely important for gut homeostasis. Accordingly, the cecal mucosa might be the most sensitive region for sensing luminal chemicals, including *ε*‐viniferin.

### Mucosal *ε*‐viniferin–evoked increase in FD4 *P* ^m→s^

**Figure 3:**
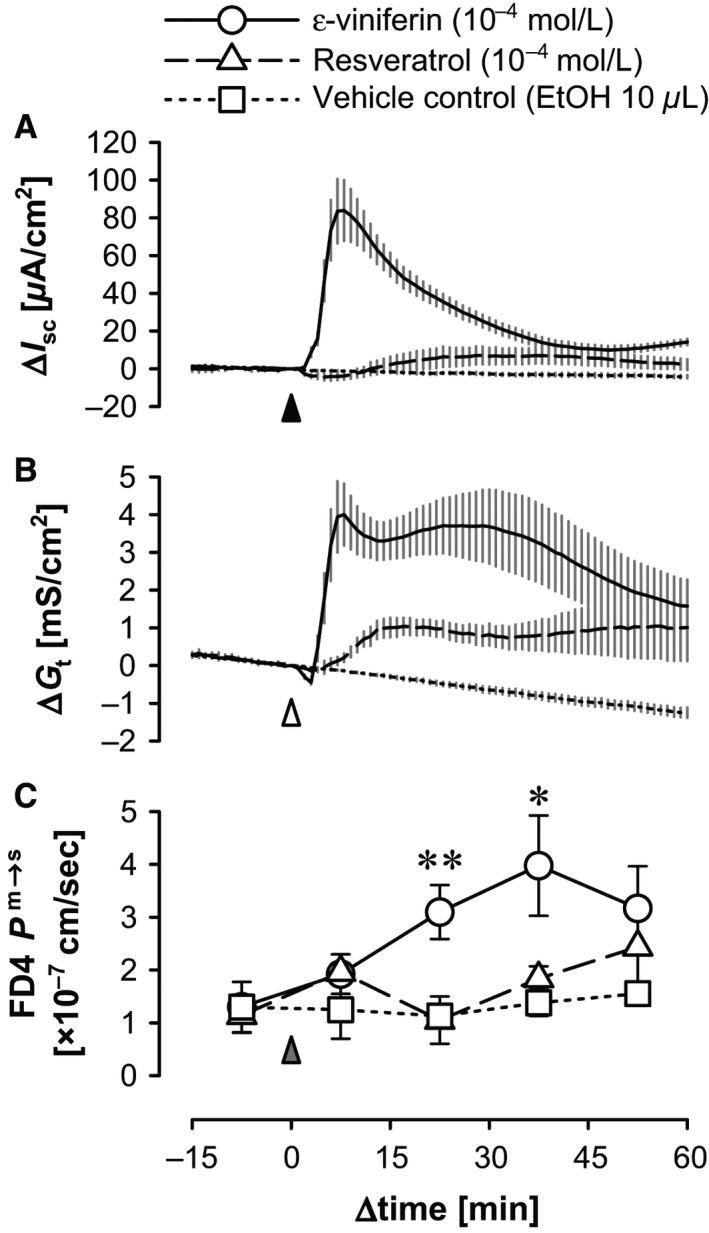
Time courses of mucosal *ε*‐viniferin– and resveratrol‐evoked changes in *I*_sc_, *G*_t_, and FD4 *P* ^m→s^ in the rat cecum. Mucosa–submucosal tissue preparations of the rat cecum were mounted on Ussing chambers, and *I*_sc_ (A) and *G*_t_ (B) were continuously recorded. FITC‐dextran (FD4; 3 kDa; 10^−4^ mol/L) was added to the mucosal bathing solution 30 min before the addition of *ε*‐viniferin (10^−4^ mol/L; *solid traces*), resveratrol (10^−4^ mol/L; *dashed traces*), and EtOH (10 *μ*L; as vehicle control: *dotted traces*). Samples (100 *μ*L) were taken from the serosal bathing solution and replaced with fresh Krebs–Ringer (100 *μ*L) every 15 min. The concentrations of FD4 in the serosal bathing solution were measured, and FD4 *P* ^m→s^ was calculated for every 15‐min time period in preparations incubated with *ε*‐viniferin (○, *solid line*), resveratrol (∆, *dashed line*), or the vehicle control (□, *dotted line*) (C). The time courses of *I*_sc_ (A) and *G*_t_ (B) every 1 min, and FD4 *P* ^m→s^ (C) in each 15‐min period are expressed as the mean ± SEM (*n *=* *5–8). *Arrow heads* indicate mucosal addition of the stimulants. *P <* *0.05 and **P <* *0.001 versus the vehicle control (Dunnett's test).

Mucosal *ε*‐viniferin (10^−4^ mol/L), but not resveratrol (10^−4^ mol/L), elicited a transient (15–45 min after addition) increase in FD4 *P* ^m→s^ (Fig. 3). In our previous study, mucosal addition of thymol also increased *I*_sc_, *G*_t_, and FD4 *P* ^m→s^ in the rat colon (Kaji et al. 2011a). In the present study, *ε*‐viniferin (10^−4^ mol/L) also increased FD4 *P* ^m→s^ in the rat cecum. These findings suggest that luminal *ε*‐viniferin directly and/or indirectly affects epithelial tight junctions, and increases paracellular permeability of nonionic macromolecules. However, the mechanism underlying the *ε*‐viniferin–evoked increase in FD4 *P* ^m→s^ needs to be examined in future studies.

### Identification of ion species involved in the mucosal *ε*‐viniferin–induced changes in *I*_sc_ and *G*_t_

**Figure 4:**
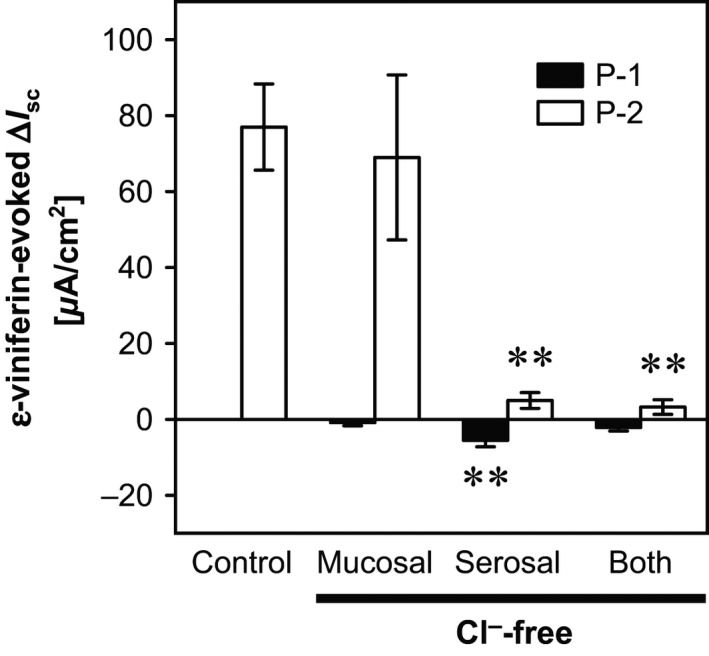
Effects of Cl^−^‐free solution on the serosal, mucosal, and both sides of the chamber on *ε*‐viniferin–evoked changes in *I*_sc_ in the rat cecum. The bathing solutions on the serosal, mucosal, and both sides were changed to Cl^−^‐free solution 30 min before the mucosal addition of *ε*‐viniferin, and the *ε*‐viniferin (10^−4^ mol/L)–evoked changes in *I*_sc_ and *G*_t_ were measured. Data are expressed as the mean ± SEM (*n *=* *4). **P <* *0.01 versus the control group (Dunnett's test).

**Figure 5:**
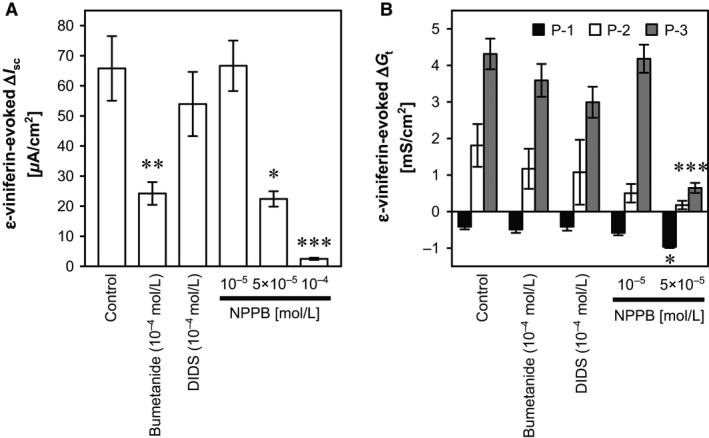
Effects of bumetanide, DIDS, and NPPB on the *ε*‐viniferin–evoked changes in *I*_sc_ and *G*_t_ in the rat cecum. *ε*‐Viniferin (10^−4^ mol/L) was added to the mucosal bathing solution in the Ussing chambers 30 min after the addition of serosal bumetanide (10^−4^ mol/L), mucosal DIDS (10^−4^ mol/L), or mucosal NPPB (10^−5^ to 10^−4^ mol/L), and the *ε*‐viniferin–induced peak changes in *I*_sc_ (A) and *G*_t_ (B) were measured. Δ*G*_t_ was not determined in the presence of 10^−4^ mol/L of NPPB, because *G*_t_ continuously increased in these conditions. Data are expressed as the mean ± SEM (*n *=* *3–7). *P <* *0.05, **P <* *0.01, and ***P <* *0.001 versus the control group (Dunnett's test).

The *ε*‐viniferin–induced increase in *I*_sc_ was due to transepithelial Cl^−^ secretion, because these responses were attenuated by Cl^−^‐free solution on the serosal and both sides, but not on the mucosal side (Fig. 4A and B). It has been proposed that intestinal fluid secretion is induced by transepithelial electrogenic anion, especially Cl^−^, secretion elicited by the collaborative functions of basolateral and apical transporters and channels (Karaki and Kuwahara 2004). In particular, the basolateral NKCC1 cotransporter promotes Cl^−^ uptake from the basolateral membrane, whereas anion channels, especially CFTR channels in the apical membrane, release anions, especially Cl^−^, into the luminal side (Karaki and Kuwahara 2004). The present results indicate that the mucosal *ε*‐viniferin–evoked positive ∆*I*_sc_ was attenuated by serosal bumetanide, a NKCC inhibitor, and by mucosal NPPB, a cAMP‐dependent Cl^−^ channel (including CFTR) blocker (Fig. 5). Accordingly, the *ε*‐viniferin–evoked positive ∆*I*_sc_ may be driven by Cl^−^ secretion. In the serosal Cl^−^‐free condition, mucosal *ε*‐viniferin evoked a negative ∆*I*_sc_, as did resveratrol (Fig. 5). This suggests that the initial *ε*‐viniferin–evoked negative ∆*I*_sc_ was masked in normal conditions. When both sides contained Cl^−^‐free solutions, *ε*‐viniferin did not evoke significant negative ∆*I*_sc_ (Fig. 5). This suggests that the initial *ε*‐viniferin–evoked negative ∆*I*_sc_, which was masked in normal conditions, is due to the presence of mucosal Cl^−^ and might be based on electrogenic Cl^−^ absorption. However, little is known about electrogenic Cl^−^ absorption in the mammalian intestine. Therefore, further studies are necessary to clarify the mechanism involved in the initial *ε*‐viniferin– or resveratrol‐evoked negative ∆*I*_sc_.

Although the *ε*‐viniferin–evoked P‐1, P‐2, and P‐3 ∆*G*_t_ were not significantly affected by serosal bumetanide (10^−4^ mol/L) and apical DIDS (10^−4^ mol/L), the apical administration of NPPB at concentrations ≥5 × 10^−5^ mol/L significantly enhanced P‐1 and significantly attenuated P‐3 ∆*G*_t_ (Fig. 5B). The enhancement of the *ε*‐viniferin–evoked P‐1 ∆*G*_t_ by NPPB might be shown by the NPPB‐mediated abolishment of the *ε*‐viniferin–evoked increase in *G*_t_. The *ε*‐viniferin–evoked change in *G*_t_ was likely due to the change in the condition of tight junctions. However, the mechanism by which mucosal NPPB inhibited the *ε*‐viniferin–evoked P‐2 and P‐3 ∆*G*_t_ was unclear, so further studies are need to clarify the mechanism underlying the *ε*‐viniferin–evoked changes in *G*_t_.

### Antagonistic effects of resveratrol on *ε*‐viniferin–evoked changes in *I*_sc_ and *G*_t_

**Figure 6:**
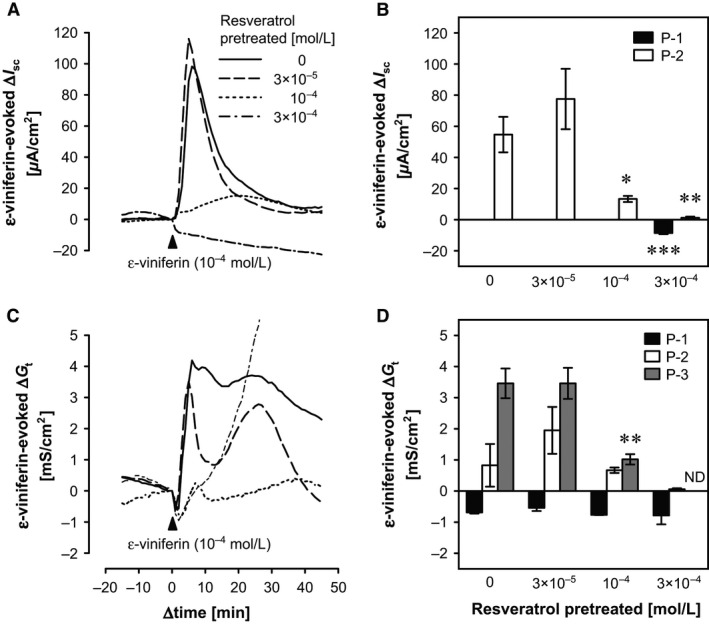
Effects of mucosal resveratrol on the mucosal *ε*‐viniferin–evoked changes in *I*_sc_ and *G*_t_ in the rat cecum. Representative traces of the mucosal *ε*‐viniferin (10^−4^ mol/L)–evoked changes in *I*_sc_ and *G*_t_ in the presence of mucosal resveratrol (0 mol/L and 3 × 10^−5^ to 3 × 10^−4^ mol/L) are shown in (A) and (C), respectively, and the mean ± SEM (*n *=* *4–5) values were shown in (B) and (D), respectively. In the presence of 3 × 10^−4^ mol/L resveratrol, the *ε*‐viniferin–evoked Δ*G*_t_ was not determined (ND), because the *G*_t_ continuously increased in these conditions. *P <* *0.05 and **P <* *0.001 versus the control group (Dunnett's test).

Although mucosal resveratrol at 10^−4^ mol/L hardly affected *I*_sc_ or *G*_t_, it inhibited the mucosal *ε*‐viniferin–evoked increases in *I*_sc_ and the increase in *G*_t_ (Fig. 6). This suggests that 10^−4^ mol/L resveratrol has antagonistic effects against the receptors for *ε*‐viniferin on the apical membrane of the epithelium without causing a tissue response. However, mucosal resveratrol did not inhibit the initial *ε*‐viniferin–evoked decrease in *G*_t_ (Fig. 6C and D). These results suggest that the *ε*‐viniferin–evoked increase and decrease in *G*_t_ are mediated via different mechanisms.

Moreover, we hypothesize that the apical membrane of cecal epithelial cells express some receptors, on which *ε*‐viniferin acts as an agonist, and resveratrol acts as an antagonist at ≤10^−4^ mol/L or as an agonist at >10^−4^ mol/L. However, the identity of these receptors and the molecular mechanisms involved in the binding of *ε*‐viniferin and resveratrol to these receptors are still unknown. Future studies should address these questions.

### The mucosal *ε*‐viniferin–evoked increase in *I*_sc_ and P‐2 increase in *G*_t_ was mediated via TRPA1

**Figure 7:**
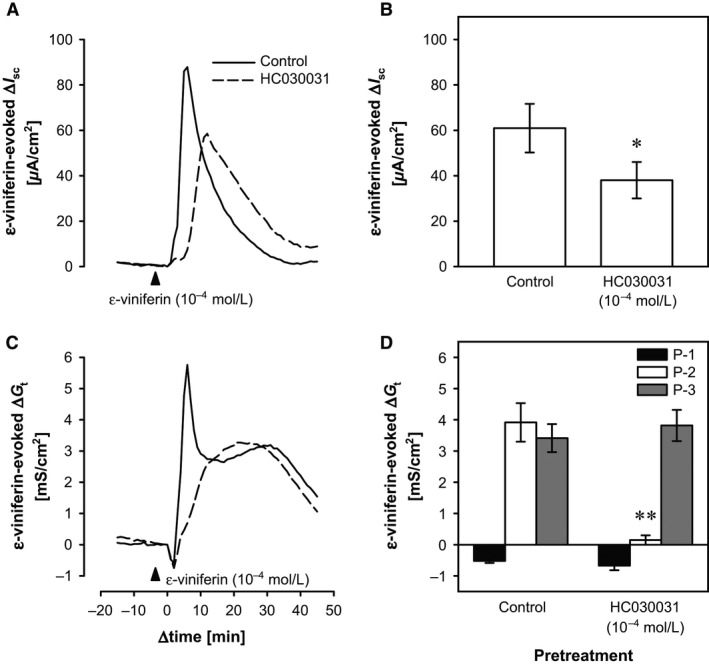
Effects of a TRPA1 inhibitor, HC030031, on the *ε*‐viniferin–evoked changes in *I*_sc_ and *G*_t_ in the rat cecum. Representative traces of the mucosal *ε*‐viniferin (10^−4^ mol/L)‐evoked changes in *I*_sc_ and *G*_t_ in the presence and absence of mucosal HC030031 (10^−4^ mol/L) are shown in (A) and (C), respectively, and the mean ± SEM (*n *=* *4) values are shown in (B) and (D), respectively. *P <* *0.05 and **P <* *0.001 versus the control group (paired *t* test).

We previously reported that a luminal stimulant, AITC, a TRPA1 agonist, induced anion secretion via EP_4_ receptors (Kaji et al. 2012). Therefore, the *ε*‐viniferin–evoked Cl^−^ secretion following the release of PGs may occur via the same signaling pathway to the AITC‐evoked response. The peak ∆*I*_sc_ of the *ε*‐viniferin–evoked increase in *I*_sc_ was significantly, but not completely, inhibited by a TRPA1 blocker, HC030031, and the peak time after the addition of *ε*‐viniferin was significantly delayed in the presence of HC030031 (Fig. 7A and B). Moreover, the *ε*‐viniferin–evoked P‐2 ∆*G*_t_ was completely abolished by HC030031 (Fig. 7C and D). Therefore, the *ε*‐viniferin–evoked increase in *I*_sc_ was partially due to TRPA1 and the increase in P‐2 ∆*G*_t_ was completely due to TRPA1. There are currently no reports showing that *ε*‐viniferin activates TRPA1, but mucosal *ε*‐viniferin may activate apical TRPA1 expressed on epithelial cells (Kaji et al. 2012).

### Mechanisms involved in mucosal *ε*‐viniferin–evoked Cl^−^ secretion and changes in ion permeability

**Figure 8:**
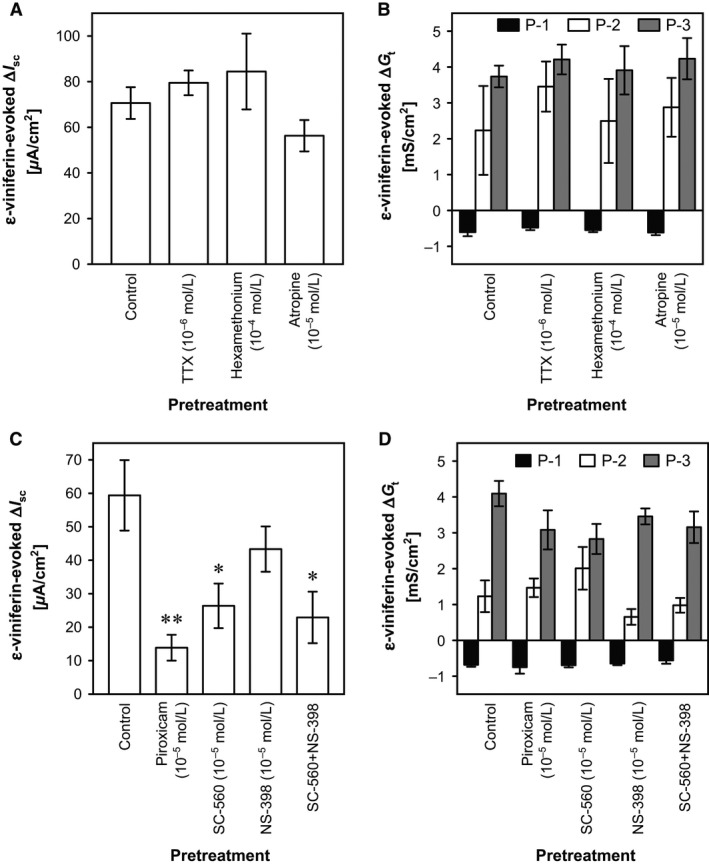
Effects of neural blockade, cholinergic antagonists, and COX inhibitors on the *ε*‐viniferin–evoked changes in *I*_sc_ and *G*_t_ in the rat cecum. *ε*‐Viniferin (10^−4^ mol/L) was added to the mucosal bathing solution 30 min after the serosal addition of a neural blocker (TTX; 10^−6^ mol/L), nicotinic acetylcholine receptor antagonist (hexamethonium; 10^−4^ mol/L), or muscarinic AChR antagonist (atropine; 10^−5^ mol/L), and the *ε*‐viniferin–evoked changes in *I*_sc_ (A) and *G*_t_ (B) were measured. The effects of a nonselective COX inhibitor (piroxicam; 10^−4^ mol/L), a selective COX‐1 inhibitor (SC‐560; 10^−5^ mol/L), a selective COX‐2 inhibitor (NS‐398; 10^−5^ mol/L), or both SC‐560 (10^−5^ mol/L) and NS‐398 (10^−5^ mol/L) on the *ε*‐viniferin–evoked changes in *I*_sc_ (C) and *G*_t_ (D) were also determined. Data are expressed as the mean ± SEM (*n *=* *3–6). *P <* *0.05 and **P <* *0.001 versus the control group (Dunnett's test).

**Figure 9:**
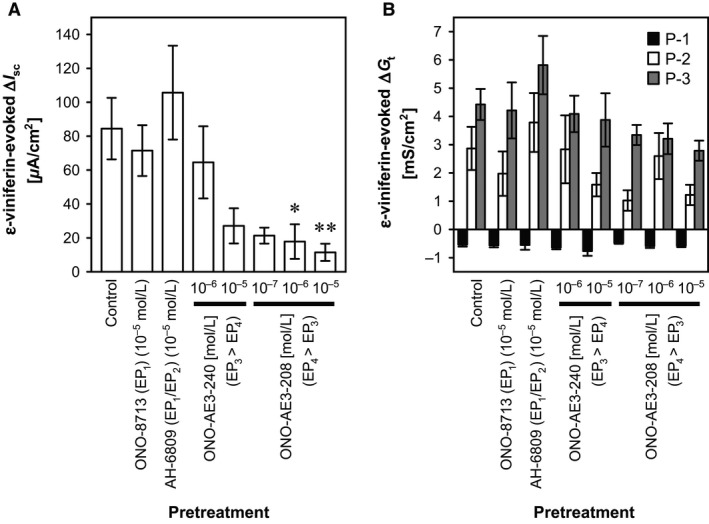
Effects of PGE_2_ receptor antagonists on the *ε*‐viniferin–evoked changes in *I*_sc_ and *G*_t_ in the rat cecum. *ε*‐Viniferin (10^−4^ mol/L) was added to the mucosal bathing solution 30 min after the serosal addition of a selective EP_1_ receptor agonist (ONO‐8713; 10^−5^ mol/L), a selective EP_1_/EP_2_ receptor antagonist (AH‐6809; 10^−5^ mol/L), an EP_3_ receptor antagonist (EP_3_ > EP_4_; ONO‐AE3‐240; 10^−6^ and 10^−5^ mol/L), an EP_4_ receptor antagonist (EP_4_ > EP_3_; ONO‐AE3‐208; 10^−7^ to 10^−5^ mol/L), or EtOH as a vehicle control (10 *μ*L), and the *ε*‐viniferin–evoked peak changes in *I*_sc_ (A) and *G*_t_ (B) were measured. Data are expressed as the mean ± SEM (*n *=* *3–10). *P <* *0.05 and **P <* *0.01 versus the control group (Dunnett's test).

**Figure 10:**
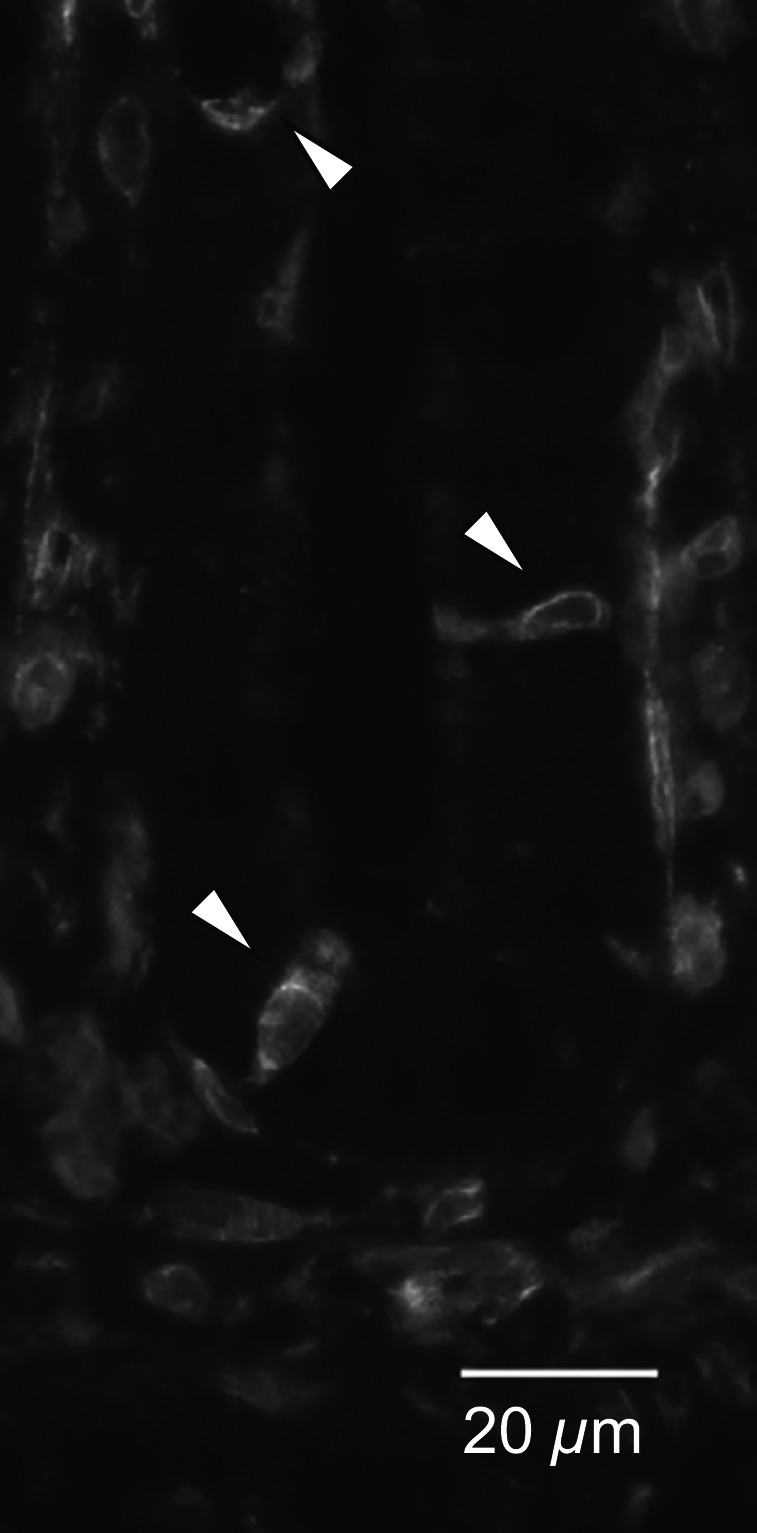
COX‐1 immunohistochemistry in the rat cecum. Four‐*μ*m thick cryostat sections of fresh cecal tissues were fixed with cold methanol, and immunostained with a goat anti‐COX‐1 primary antibody and a donkey anti‐goat IgG antibody conjugated to Alexa594. COX‐1 immunoreactive crypt cells in the rat cecum are indicated by arrowheads.

The present results suggest that *ε*‐viniferin–evoked Cl^−^ secretion is due to the production of PG by COX‐1, and that the *G*_t_ response is independent of PG (Fig. 8C). ONO‐AE3‐208, a selective EP_4_ (EP_4_ > EP_3_) antagonist, significantly attenuated *ε*‐viniferin–evoked Cl^−^ secretion (Fig. 9A). Therefore, it appears that luminal *ε*‐viniferin stimulates COX‐1 activity and PG production in sensory epithelial cells in the cecum. An earlier study revealed that COX‐1–expressing epithelial cells are scattered throughout the crypts in the rat colon (Shao et al. 1999), and we confirmed the existence of these cells in the rat cecal epithelium by immunohistochemistry (Fig. 10). Accordingly, it appears that PGs activate EP_4_ receptors on the secretory epithelial cells, ultimately inducing Cl^−^ secretion. It has been reported that EP_2_ and EP_4_ receptors, but not EP_1_ and EP_3_ receptors, mediate PGE_2_‐evoked Cl^−^ secretion via the cAMP pathway (Mosa et al. 2008).

In addition, the *ε*‐viniferin–evoked changes in ion permeability did not involve neural pathways or PG‐related pathways. Therefore, it seems likely that the *ε*‐viniferin–evoked changes in ion permeability occurred through direct effects of *ε*‐viniferin on the epithelial cells.

### Inhibitory effects of mucosal *ε*‐viniferin on mucosal propionate‐evoked Cl^−^ secretion

**Figure 11:**
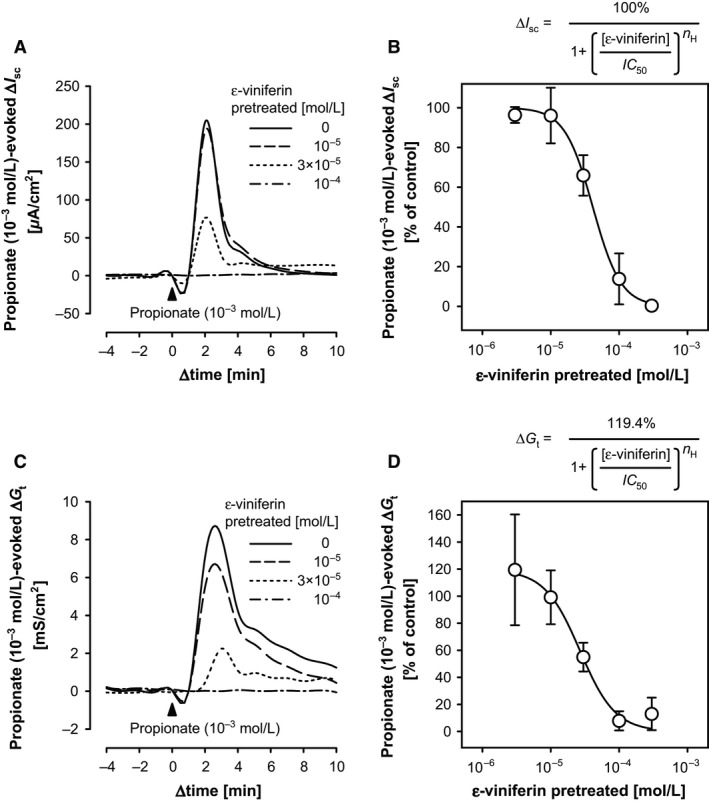
Inhibitory effects of *ε*‐viniferin on the mucosal propionate‐evoked changes in *I*_sc_ and *G*_t_ in the rat cecum. Sodium propionate (10^−3^ mol/L) was added to the mucosal bathing solution (▲) 1 h after the mucosal addition of *ε*‐viniferin (3 × 10^−6^ to 3 × 10^−4^ mol/L) or the vehicle control (10 *μ*L of EtOH; Δ). Representative traces of the propionate‐evoked increase in *I*_sc_ and *G*_t_ in the presence (10^−5^ to 10^−4^ mol/L) of *ε*‐viniferin or the vehicle control are shown in (A) and (B), respectively. The percent changes relative to the control group were plotted and fitted using the nonlinear square procedure to the inhibitory Hill equation. Data are expressed as the mean ± SEM (*n *=* *3–6).

Bacterial fermentation in the lumen of the large intestine produces numerous metabolites. The predominant molecules are SCFAs, particularly acetate, propionate, and butyrate. Propionate and butyrate, but not acetate, were reported to induce anion secretion in the rat (Yajima 1988) and guinea pig (Karaki and Kuwahara 2011) colon. Therefore, some molecules, which modulate the effects of SCFAs in the colon, critically affect the physiologic and/or pathophysiologic conditions of the large intestine. The present study showed that mucosal *ε*‐viniferin attenuated the luminal propionate (1 mmol/L)‐evoked increase in *I*_sc_ and *G*_t_ in a concentration‐dependent manner (Fig. 11). This indicates that *ε*‐viniferin does not inhibit the secretory functions of epithelial cells, but instead suggests that *ε*‐viniferin might affect the mechanism for sensing propionate. Although the mechanism by which *ε*‐viniferin may inhibit the propionate‐evoked *I*_sc_ response is unclear, the data suggest that the inhibitory effects of *ε*‐viniferin on the propionate‐evoked responses are mediated by positive cooperative binding because *n*_H_ was >1. The propionate‐evoked response is thought to be mediated by its receptors, namely free fatty acid receptor 2 (FFA2 or GPR43) and/or FFA3 (GPR41) (Karaki et al. 2006, 2008; Tazoe et al. 2009; Karaki and Kuwahara 2011). Thus, *ε*‐viniferin may allosterically bind to these receptors, with a possible stoichiometry of 2:1 because the *n*_H_ was nearly 2. Nevertheless, further studies are necessary to confirm this hypothesis. Moreover, at the lower concentration of 3 × 10^−6^ mol/L, *ε*‐viniferin very weakly enhanced the propionate‐evoked increase in *G*_t_ (Fig. 11C). Therefore, the effects of lower concentrations of *ε*‐viniferin on transepithelial ion permeability need to be investigated in future studies.

### Physiologic relevance of sensing and responding to mucosal *ε*‐viniferin in the intestinal mucosa

In the gastrointestinal lumen, especially in the large intestine, the luminal microbiota can synthesize a variety of compounds and some of these compounds may have cytotoxic effects. Thus, the intestinal mucosa has protective roles, including enhancing the integrity of the epithelial barrier and fluid secretion when the mucosa senses potentially cytotoxic chemicals. We hypothesized that the tissue concentrations of PGs may constitute an alarm‐response system, in which an increase in the PG concentration might enhance the protective functions of the mucosa in host defense (Karaki and Kuwahara 2004). Therefore, *ε*‐viniferin may activate COX‐expressing cecal crypt cells, increase the tissue PG level as part of a tissue alarm‐response system, and enhance the tissue's host defense functions.

## Conclusion

The present study shows that administration of *ε*‐viniferin to the mucosal side of the rat intestine modulates transepithelial ion transport, ion permeability, and the permeability of nonionic macromolecule as well as the effects of the other luminal molecules, such as SCFAs. These results also imply that *ε*‐viniferin has beneficial effects on intestinal functions by enhancing the mucosal host defense mechanism.

## Conflict of Interest

A. Kuwahara have received research fund from FANCL Corporation. I. Ishikawa is an employee of FANCL Corporation.
